# A novel small compound TOIDC suppresses lipogenesis via SREBP1-dependent signaling to curb MAFLD

**DOI:** 10.1186/s12986-022-00713-0

**Published:** 2022-12-06

**Authors:** Yaodi Shao, Zhi Yao, Junyi Zhou, Miao Yu, Suzhen Chen, Yanmei Yuan, Liu Han, Liqin Jiang, Junli Liu

**Affiliations:** 1 Shanghai Key Laboratory of Diabetes Mellitus, Shanghai Diabetes Institute, Department of Endocrinology and Metabolism, Shanghai Sixth People’s Hospital Affiliated to Shanghai Jiao Tong University School of Medicine, Shanghai, 200233 China; 2 School of Chemistry and Molecular Engineering, East China Normal University, Shanghai, 200241 China

**Keywords:** Metabolic-associated fatty liver disease, High content screening, Pharmacotherapy, Sterol regulatory element-binding protein 1, De novo lipogenesis

## Abstract

**Background:**

Inhibition of hepatic lipogenesis is widely regarded as an effective treatment for metabolic-associated fatty liver disease (MAFLD), although numerous related drugs have failed to reach clinical application. The goal of this study is to identify a novel small compound that can effectively treat MAFLD.

**Methods:**

Primary hepatocytes were first exposed to palmitic acid and oleic acid, then treated with compounds prior to high through screening for cellular lipid content. The efficacy of these compounds was measured by Nile Red staining and triglyceride analysis. The potential cellular toxicity caused by these compounds was evaluated by CCK8 assay. qPCR and Western blot were used to determine expression of RNAs and proteins, respectively. The compound was intraperitoneally injected into diet-induced obese (DIO) mice to examine its efficacy in vivo.

**Results:**

We identified the dimethyl 1-methyl-2-thioxoindoline-3,3-dicarboxylate (TOIDC) as a powerful chemical to reduce cellular lipid with minimal cellular toxicity. When injected intraperitoneally, TOIDC effectively ameliorates MAFLD in DIO mice. Mechanically, TOIDC suppresses de novo lipogenesis through inhibiting sterol regulatory element-binding protein 1 (SREBP1).

**Conclusions:**

Our findings indicate that TOIDC could be a promising lead compound to develop new drugs to treat MAFLD.

**Graphical Abstract:**

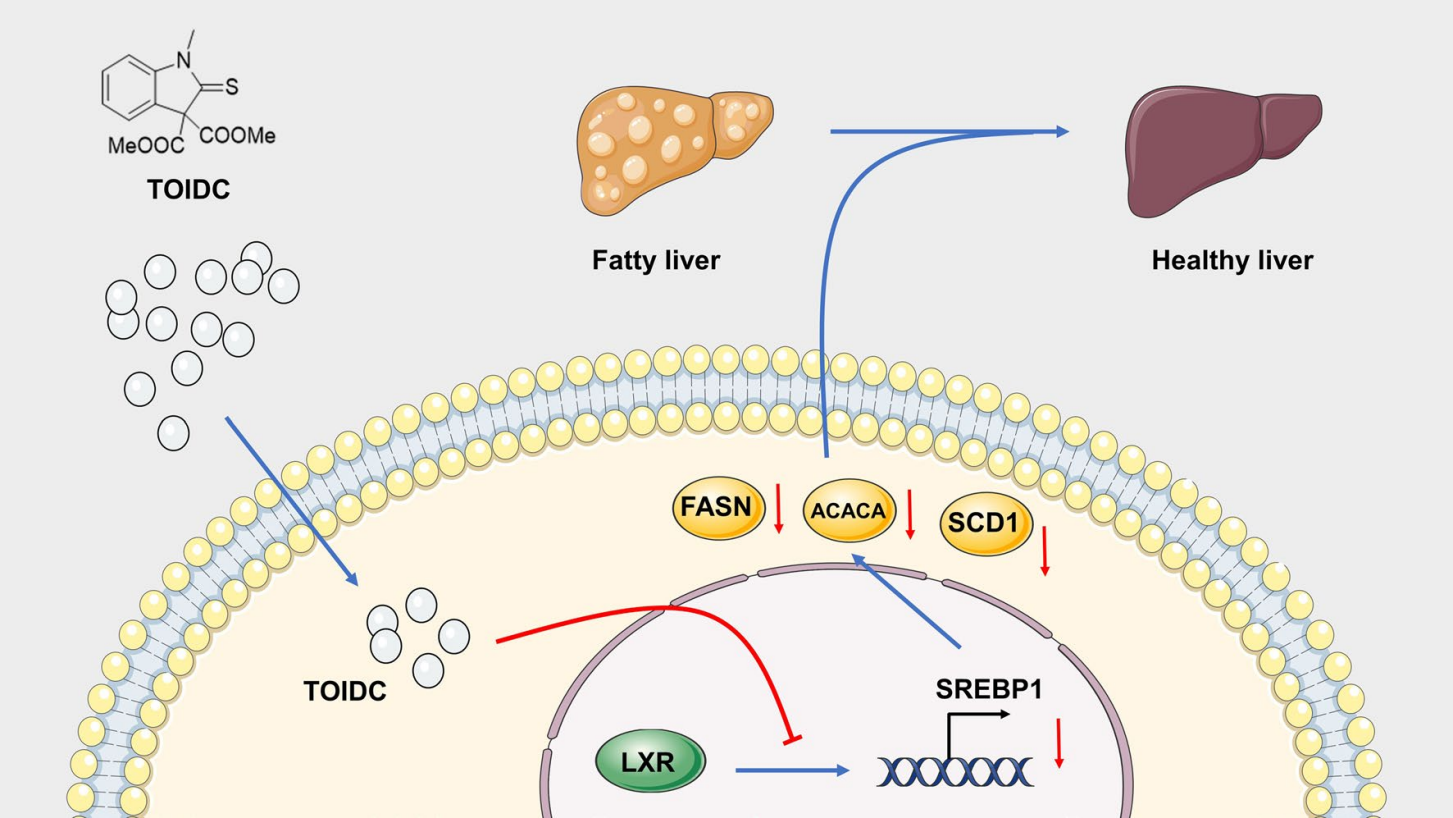

**Supplementary Information:**

The online version contains supplementary material available at 10.1186/s12986-022-00713-0.

## Introduction

Metabolic-associated fatty liver disease (MAFLD) is a serious epidemical disease influencing about 25% of the population in the world and it is receiving an increasing amount of attention [1–3]. MAFLD is defined as the dysfunctional metabolic disease with ectopic excessive 5.5% triglycerides (TG) in the liver and at least one of the three criteria, which includes overweight or obesity, type 2 diabetes mellitus (T2DM), or clinical evidence of metabolic dysfunction, such as an increased waist circumference and an abnormal lipid or glycemic profile [4]. Unfortunately, the majority of MAFLD patients are asymptomatic until an advanced stage. Furthermore, the lack of clinical drugs to inhibit the progression puts patients at a higher risk of developing carcinoma. Notably, MAFLD could be more easily reversed at an earlier period, therefore it is critical to find effective drugs to inhibit the progression of MAFLD at an early stage [5, 6]. It has been reported that the lipotoxicity induced by excessive free fatty acids (FFAs) and intermediates plays a major role in liver injury, which eventually leads to MAFLD [7]. Hepatic FFAs come from two main sources. First, FFAs are decomposed from adipose tissue or transported from small intestine which absorbs dietary nutrition [8]. Second, the hepatic de novo lipogenesis (DNL) pathway can also produce FFAs in the liver. However, some studies have reported that in people whose liver accumulates TG, the levels of newly synthesized VLDL-TG are twice as high as those of general people, suggesting that DNL is the primary source of FFAs [9]. Additionally, DNL not only increases FFAs, but also inhibit β-oxidation through its intermediate products,, which further aggravates MAFLD [10].

Sterol regulatory element-binding proteins (SREBPs) are major transcription factors that regulate lipid homeostasis mainly via cholesterol and fatty acid metabolism [11, 12]. SREBPs have three isoforms, including SREBP2, SREBP1a, and SREBP1c. Cleaved SREBP1 (SREBP1c) is abundant in the liver. SREBP1c is transported into the nucleus after being cleaved where it generally initiates the DNL pathway [13, 14]. Clinically, various studies have reported that inhibition of the DNL pathway is beneficial to ameliorate MAFLD [15, 16]. Based on these reports, various drugs are under research, targeted at the key rate-limiting enzymes of the DNL pathway. For instance, Firsocostat is an inhibitor of acetyl-CoA carboxylase (ACACA), and TVB 2640 inhibits fatty acid synthesis (FASN) enzyme [17, 18]. However, none of these compounds have been approved for clinical application due to their side effects such as a higher risk of hypertriglyceridemia. Therefore, relying on DNL pathway suppression to ameliorate MAFLD is still challenging.

In this study, we have screened a series of small molecules by high-content screening (HCS). Intriguingly, dimethyl 1-methyl-2-thioxoindoline-3,3-dicarboxylate (TOIDC) has been found to effectively reduce lipid accumulation in vitro with minimal toxicity. TOIDC is a novel compound derived from 2-thioxoindoline scaffold, which exhibits a variety of biological activity; nevertheless, TOIDC is the only 2-thioxoindoline-derivated drug that ameliorates MAFLD, compared to other chemical containing 2-thioxoindoline structure. Adding ester group to TOIDC increases its lipophilicity, which has more benefits to exert its biological activity in the cells. Surprisingly, additional experiments showed that TOIDC not only ameliorates lipid accumulated in the liver but also reduces subcutaneous fat in vivo with no disruption of food intake. In addition, TOIDC has no risk of hyperlipidemia, different with other drugs targeting MAFLD [19, 20]. Mechanically, we demonstrate that TOIDC ameliorates MAFLD by reducing SREBP1 expression that in turn decreases the expression of lipid synthesis genes, including Fasn, Acaca, and Scd1. Therefore, these findings indicate that TOIDC might be useful for exploring new target or optimal drug for MAFLD.

## Materials and methods

### Compounds

All chemical compounds were synthesized by the laboratory of Prof. Jiang from East China Normal University (Shanghai, China) and were dissolved in dimethyl sulfoxide (DMSO) prior to use.

### Mouse models

Four-week-old, body weight 18 ± 1 g, male C57BL/6 J mice were purchased from Shanghai SLAC Laboratory Animal Co., Ltd. (Shanghai, China) and housed in the specific pathogen free (SPF) facility under controlled environmental conditions (temperature 24–26 °C; relative humidity 60–70%). After importing into the facility, all mice were acclimatized to their environment for two weeks. For generating diet-induced obese (DIO) mice, six-week-old mice were given a high-fat diet (HFD) from Research Diets, Inc. (New Brunswick, NJ, USA) for a total period of 16 weeks before experiments. The DIO mice were randomly divided into two groups (n = 5/group) and were intraperitoneal injected (i.p.) with TOIDC (10 mg/kg body weight) or vehicle (DMSO) once daily for weeks. All animal studies were conducted in accordance with the Provision and General Recommendation of Chinese Experimental Animals Administration Legislation, and the study procedures were approved by the Animal Experimentation Ethics Committee of Shanghai Jiao Tong University Affiliated Sixth People’s Hospital.

### Mouse serum assay and liver function analyses

Alanine aminotransferase (ALT) and aspartate aminotransferase (AST) levels were measured with the tail vein blood to assess liver injury using the commercial assay kit from Nanjing Jiancheng Bioengineering Institute (Nanjing, China).

### Hepatic lipid extraction and analysis

For quantification of intrahepatic lipid, tissues were homogenized with phosphate-buffered saline (PBS) and centrifuged, then the liquid phases were transferred into clean tubes. The lipid levels were determined using TG assay kit from Solarbio (Beijing, China), respectively, and normalized to the tissue weight.

### Histological analyses

Liver tissues were embedded in 4% paraformaldehyde fix solution (PFA) or OCT compound, then stained with H&E or Nile Red to visualize the lipid accumulation in liver. The histological features were observed under a light microscope and imaged.

### Glucose and insulin tolerance tests

Before glucose tolerance test (GTT), mice had free access to drinking water but were fasted 16 h. The fasting blood glucose of each mouse was measured followed by an i.p. injection of D-glucose (20% in saline, 1.5 g/kg body weight). Insulin tolerance tests (ITT) were conducted in mice fasted 4 h by i.p. injection of insulin (0.75 IU/kg body weight). The vein blood was tested at 0, 15, 30, 60, 90 and 120 min after injection.

### Cell culture

HepG2 cells were purchased from Shanghai institute of biochemistry and cell biology (Shanghai, China) and maintained in high glucose DMEM containing 10% fetal bovine serum (FBS), 100 U/ml penicillin, and 100 μg/ml streptomycin at 37 °C in a humidified atmosphere containing 5% CO2. Primary hepatocytes were isolated from 6-week-old male C57BL/6 J mice. The mice were anesthetized and perfused with perfusion buffer and collagenase-I through the portal vein at 37 °C. The lysates filtered through a 100 mm cell strainer (Thermo Fisher Scientific, Waltham, MA, USA) and spun at 400 g/min for 3 min at 4 °C. The cells were then resuspended in mixture containing 43% Percoll and 4.8% 10 × PBS and spun at 700 g/min for 10 min at 4 °C. Finally, cells were resuspended in low glucose medium (Yuanpei, Shanghai, China) and plated at the specific density. A mixture of 0.2 mM oleic acid (OA) and 0.1 Mm palmitic acid (PA) (Sigma-Aldrich, St. Louis, MO, USA) in 0.5% BSA was added into low glucose media containing only 2% FBS to establish an in vitro model of lipid accumulation in hepatocytes overnight.

### In vitro compounds treatment

To investigate the effects of the compounds, hepatocytes were treated with vehicle or the compounds for 12–36 h. For Nile Red staining, hepatocytes were incubated with a medium containing OA and PA (total 0.3 mM); after 12–18 h, cells were then incubated with vehicle or TOIDC for another 36 h. For quantification of mRNA and protein level, hepatocytes were treated with vehicle or TOIDC for 12–36 h. For the activator’s experiment, hepatocytes were treated with a mixture of TOIDC and 15 or 30 µM T0901317 (MedChemExpress, Monmouth Junction, NJ, USA) for 12–36 h.

### Viability assay

Cell viability was accessed by the CCK8 assay (Beyotime, Shanghai, China). Hepatocytes were treated with compounds at about 5 × 10^3^ cells/well in 96-well plates. After 32 h, cells were incubated with 10% CCK8 reagent for another 4 h. The cell viability was detected using micro-plate spectrophotometer at OD 450 nm.

### MitoTracker staining

The cultured medium of hepatocytes was removed and added fresh medium containing 200 nM MitoTracker Red probe (Invitrogen, Carlsbad, CA, USA) for 30 min at 37 °C, then wash the dish with PBS for 3 times and incubated with fresh culture medium. Images was captured by the fluorescence microscope.

### Immunoblotting

Liver tissues or hepatocytes lysates were extracted with radioimmunoprecipitation assay (RIPA) buffer (50 mM Tris–HCl, 50 mM NaCl, 1% NP-40, 0.1% SDS, 0.5% sodium deoxycholate 8.0) including 0.5% protease inhibitors and 1% phosphorylation inhibitors. The protein samples were loaded into a 10% SDS-PAGE gel and transferred onto a polyvinylidene difluoride membrane. After 5% bovine serum albumin (BSA) blocked for 1 h, the membrane was incubated with primary antibodies at 4 °C overnight, and then secondary antibodies for 1 h at room temperature. Immunoreactivities were detected with enhanced chemiluminescent autoradiography (Merck Millipore, Billerica, MA, USA). Chemiluminescence was determined using the AI600 System (GE Healthcare, Little Chalfont, Buckinghamshire, UK). The antibodies used for immunoblotting specific to SREBP1 (1:1000, 14,088–1-AP, Proteintech), FASN (1:1000, SC-48357, Santa Cruz), carnitine palmitoyl transferase 1 alpha (CPT1α, 1:1000, 15,184–1-AP, Proteintech), SCD1 (1:1000, SC-515875, Santa Cruz), GAPDH (1:1000, 60,004–1-Ig, Proteintech), α-Actinin (1:1000, Cat No. 11313–2-AP, Proteintech).

### qRT-PCR

Total RNA was extracted from hepatocytes or mouse liver tissues through the Trizol reagent (Invitrogen, Carlsbad, CA, USA). Then, to obtain cDNA, reverse transcription was performed with RNA using cDNA Synthesis Kit (Vazyme Biotech, Nanjing, China). The relative abundance of specific gene was normalized to 18S and analyzed by quantitative real-time PCR system (Roche, Switzerland) using SYBR green PCR mix (Vazyme Biotech, Nanjing, China). qRT-PCR primers are available in Additional file 5: Table S2.

### High content screening (HCS)

For HCS, the primary hepatocytes were washed with PBS and fixed with 4% paraformaldehyde (PFA) for 30 min at room temperature and then incubated with Nile Red buffer purchased from Yeasen (Shanghai, China) for 15 min at 37 °C, afterward the cells were washed three times with PBS and mounted with DAPI. Images were photographed in a continuous field of view autonomously and analyzed using the High content screen system (Image Xpress Micro 4, Molecular Devices, San Francisco, CA, USA).

### Molecular docking

Crystal structures of Farnesoid X receptor (FXR) were obtained from PDB database (ID: 6HL1). Then the water molecules, redundant chains, and the original ligand molecules were deleted. The three-dimensional structure of TOIDC was constructed by Chimera software (UCSF, San Francisco, CA, USA). The energy of the receptor structure was minimized using Dunbrack 2010 rotamers. Molecular docking was then performed by AutoDock Vina software to identify potential binding sites. The binding pose of TOIDC with the lowest score was chosen, and hydrogen bonds were analyzed between TOIDC and surrounding amino acid residues.

### Statistical analysis

All data are presented as means ± SE of the means (SEM). Differences between the two groups were determined by Student’s two-tailed t-test or two-way ANOVA. When more than two groups were examined, one-way ANOVA followed by Tukey's test was used. *P* < 0.05 was considered statistically significant. Statistical analysis was carried out using SPSS version 22.0 (IBM, Armonk, NY, USA).

## Results

### Identification of candidate compounds reducing lipid accumulation in hepatocytes through HCS

**Fig. 1:**
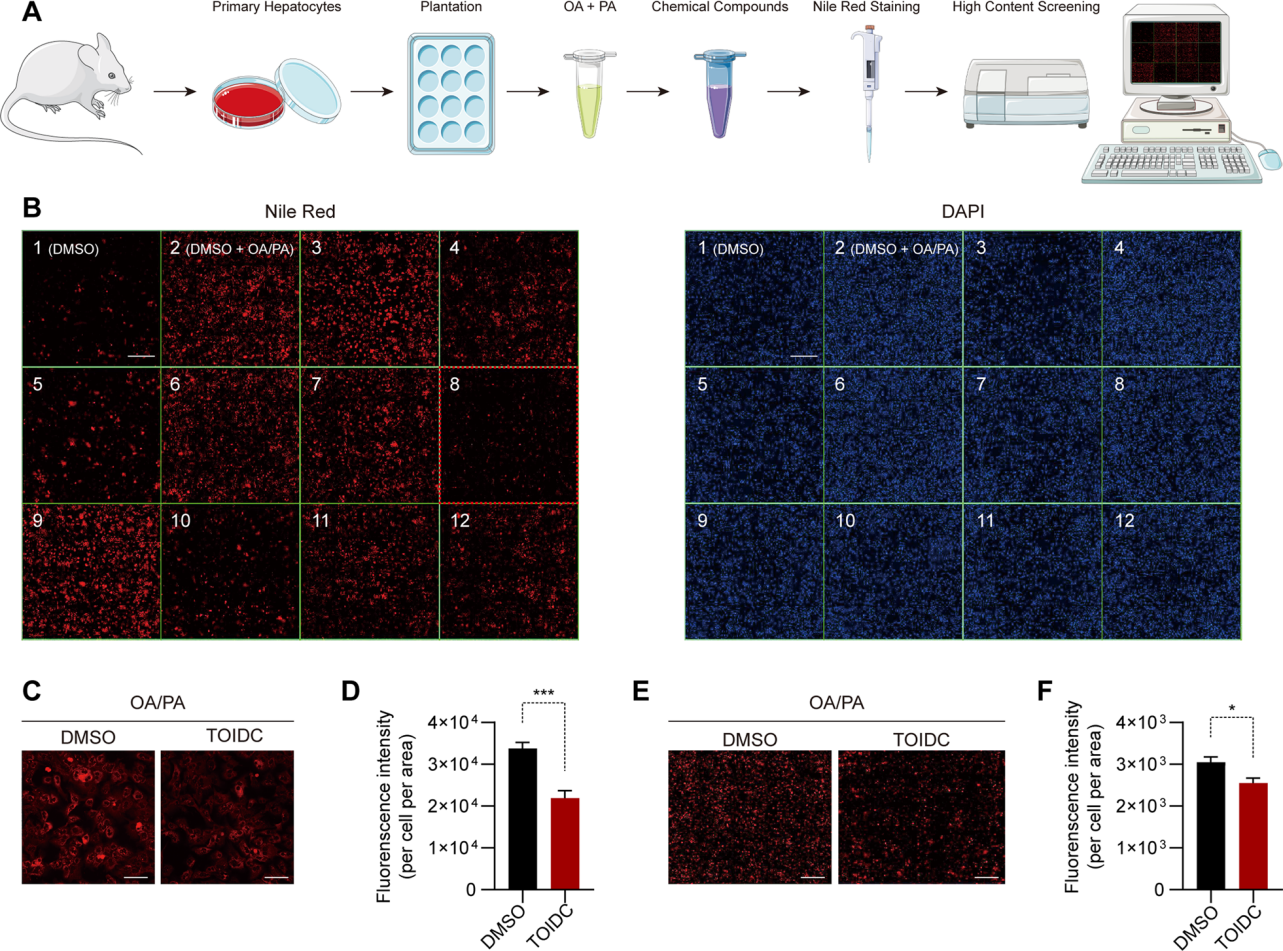
Identification of candidate compounds reducing lipid accumulation through high-content screening (HCS) analysis. **A** Flow chart depicting the process for screening the potential candidates to reduce lipid accumulation. **B** Fluorescent images representing the compounds’ effect on the lipid droplets accumulation through HCS analysis. Scale bar, 200 µm. **C** The comparison between control and TOIDC. Scale bar, 50 µm. **D** Quantification of the relative fluorescent intensity of TOIDC compared to control. **E** Fluorescent images representing the TOIDC effect on the lipid accumulation through a 6-well plate. Scale bar, 200 µm. **F** Quantification of (**E**). Data are presented as mean ± SEM of three independent biological replicates; *P < 0.05; **P < 0.01; *ns* no significance

We used HCS to perform a high-throughput and efficient screening for 98 different chemical compounds that may have potential effects on lipid accumulation in primary hepatocytes in vitro (Additional file 4: Table S1). Primary hepatocytes were isolated from 6 ~ 8 weeks-old C57BL/6 J mice and lipid accumulation was induced by incubating them in a medium containing 300 µM mixture of OA and PA overnight. The next day, the medium was replaced with a fresh medium containing various compounds (20 µM). Later, the primary hepatocytes were stained with Nile Red solution, and the fluorescence intensity was detected through HCS (Fig. 1A). Based on the HCS analysis, we identified TOIDC (No.8) dramatically reduced lipid accumulation in primary hepatocytes compared to other similar structure-like compounds (Fig. 1B–D). The experiment was repeated on primary hepatocytes seeded in 6-well plates to confirm our findings, and TOIDC showed inhibitory effects on lipid accumulation (Fig. 1E–F). Besides, we also examined the compounds derived based on TOIDC structure and found that none of them showed an effect on hepatocytes, or had lethal toxicity (Additional file 1: Figure S1A–B). These results indicated that the structure of TOIDC is optimal for further studies.

### Validation of TOIDC as a novel small compound to reduce lipid accumulation

**Fig. 2:**
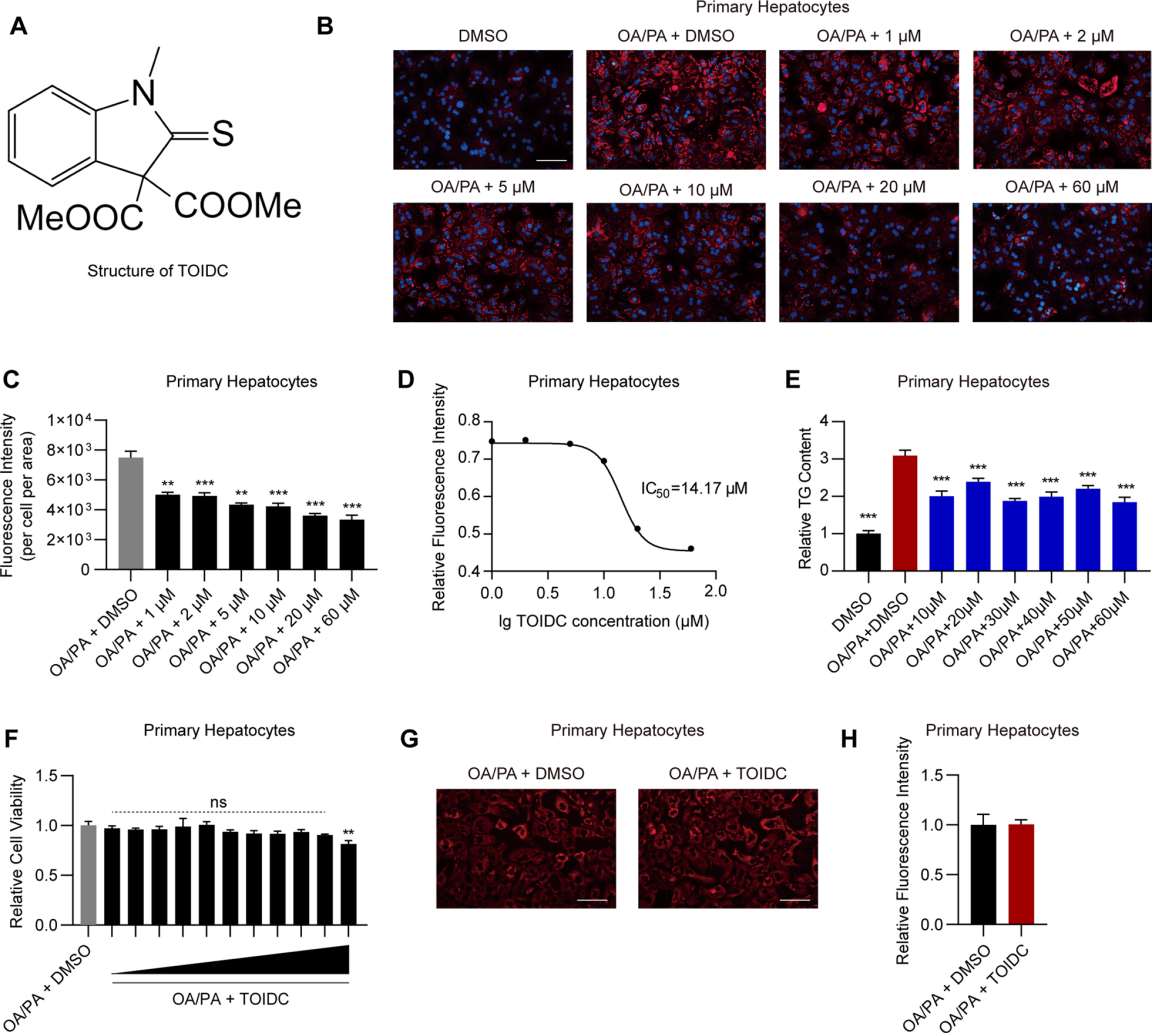
Validation of TOIDC as a novel small compound to reduce lipid accumulation. **A** Structure of TOIDC. **B** Representative Nile Red stained images of the vehicle and TOIDC-treated primary hepatocytes. Scale bar, 100 µm. **C** Quantification of (**B**). **D** IC50 curve of TOIDC in primary hepatocytes treated with oleic acid and palmitic acid. **E** Dose response of triglyceride content in the treated primary hepatocytes. **F** CCK8 assay of cell viability in TOIDC-treated primary hepatocytes. **G** Representative images of MitoTracker Red staining in primary hepatocytes treated with vehicle or TOIDC (15 µM) for 36 h. Scale bar, 50 µm. **H** Quantification of (**G**). Data are presented as mean ± SEM of three independent biological replicates; *P < 0.05; **P < 0.01; *ns* no significance

TOIDC has a novel structure compared to other drugs (Fig. 2A). Consistent with the observations obtained from HCS that TOIDC substantially reduces intracellular lipid accumulation (Fig. 1B), we further analyzed the TOIDC-treated primary hepatocytes with a fluorescence microscope and confirmed that TOIDC dose-dependently decreased lipid accumulation in mouse primary hepatocytes (Fig. 2B). In addition, we quantified the fluorescent intensity in Fig. 2B and calculated the half-maximal inhibitive concentration (IC_50_) of TOIDC to be 14.17 µM (Fig. 2C), which indicates a higher activity of TOIDC in the reduction of lipid accumulation. Interestingly, TG analysis showed that TG content was also decreased in primary hepatocytes with an increasing dose of TOIDC (Fig. 2E). Intriguingly, TOIDC showed undetectable cytotoxicity when TOIDC concentration was less than 20 µM (higher than IC50), and only mild cytotoxicity at the highest dose (60 µM) (Fig. 2F). MitoTracker staining showed no significant difference between vehicle-treated and TOIDC-treated mouse primary hepatocytes (Fig. 2G–H). These results demonstrated that the TOIDC could substantially reduce intracellular lipid accumulation without obvious cytotoxicity.

### TOIDC has a comparable effect on HepG2 similar to the primary hepatocytes

**Fig. 3:**
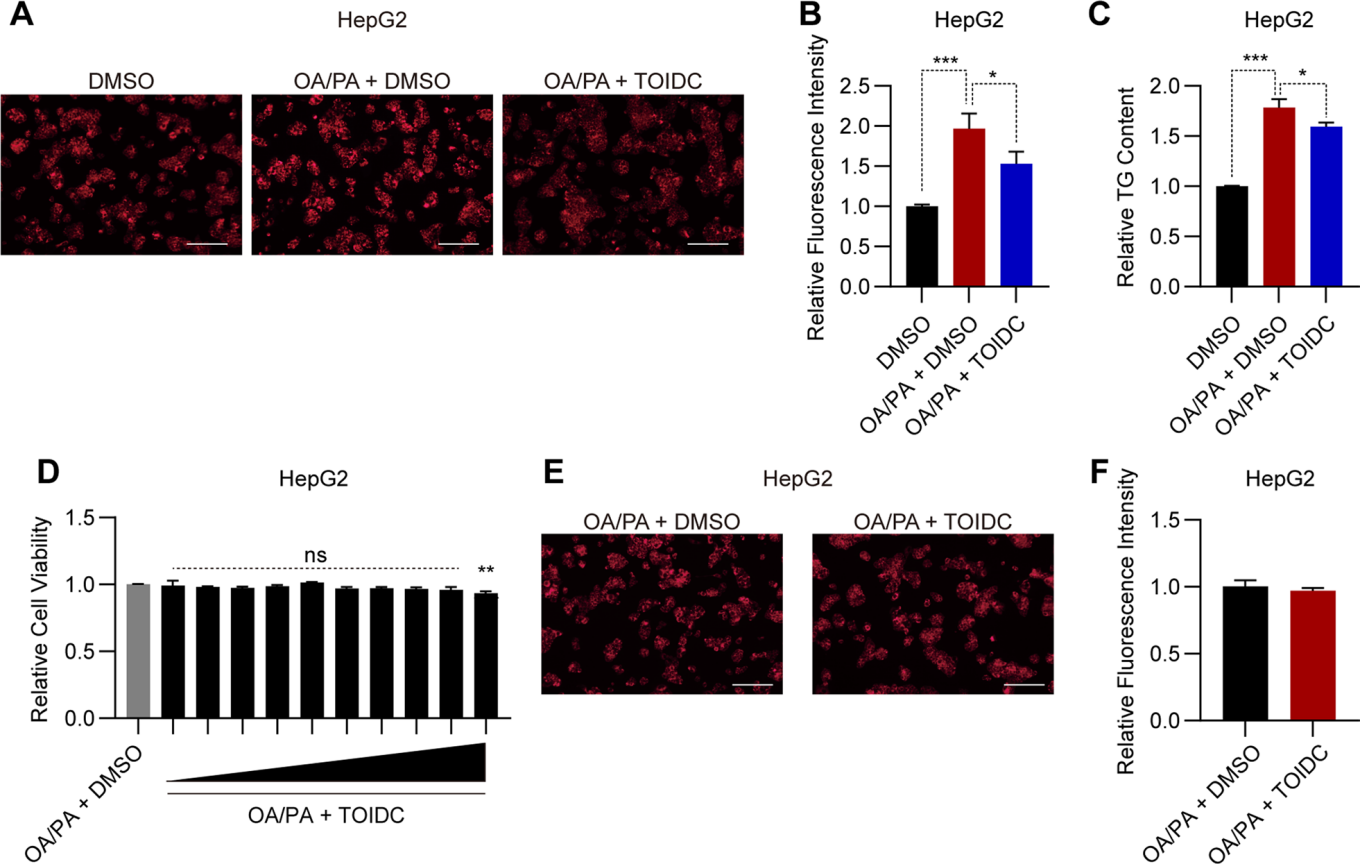
TOIDC has a comparable effect on the human-derived cells with the primary hepatocytes. **A** Representative Nile Red stained images of the vehicle and TOIDC-treated HepG2. Scale bar, 100 µm. **B** Quantification of (**A**). **C** Triglyceride content of HepG2 treated with vehicle or TOIDC. **D** CCK8 assay of cell viability in TOIDC-treated HepG2. **E** Representative images of MitoTracker Red staining in HepG2 treated with vehicle or TOIDC (15 µM) for 36 h. Scale bar, 50 µm. **F** Quantification of (**E**). Data are presented as mean ± SEM of three independent biological replicates; *P < 0.05; **P < 0.01; *ns* no significance

Next, we questioned whether TOIDC has a therapeutic effect of reducing lipid accumulation on human hepatocytes (cell line, HepG2). Consistent with previous results, TOIDC showed a strong ability to reduce lipid accumulation in HepG2 cells compared to vehicle, both by Nile Red staining (Fig. 3A–B) and TG analysis (Fig. 3C). The CCK8 assay revealed that TOIDC has no detective cytotoxicity on HepG2 (Fig. 3D). In addition, MitoTracker staining also showed no significant difference between vehicle-treated and TOIDC-treated HepG2 cells (Fig. 3E–F). These results suggested that TOIDC has comparable effects on hepatocytes obtained from both mouse and human. Additionally, these data provide us with evidence that TOIDC can significantly decrease lipid accumulation in human cell lines, which can have implications for further studies on therapeutic adaptation.

### TOIDC reduces lipid accumulation through regulation of the SREBP1-dependent lipogenesis pathway

**Fig. 4:**
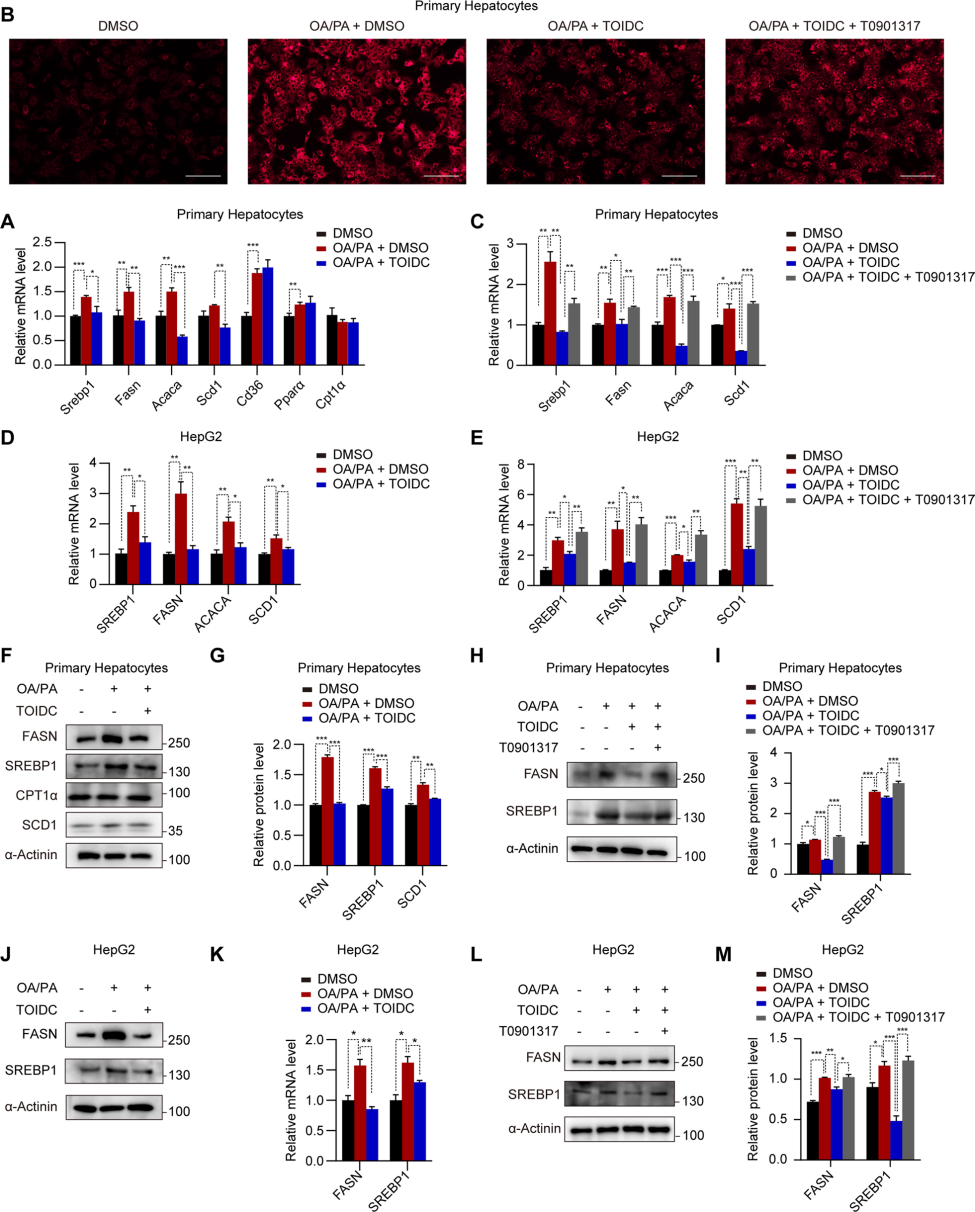
TOIDC reduces lipid accumulation through regulation of the SREBP1-dependent lipogenesis pathway. **A** Abundance of mRNAs in primary hepatocytes stimulated by oleic acid (OA) and palmitic acid (PA) previously treated with vehicle or TOIDC. **B** Representative Nile Red-stained images of the vehicle, TOIDC-treated, and T0901317-treated primary hepatocytes. Scale bar, 100 µm. **C** Abundance of lipogenesis-related mRNAs in primary hepatocytes stimulated by OA/PA previously treated with vehicle or TOIDC and T0901317. **D** Abundance of lipogenesis-related mRNAs in HepG2 stimulated by OA/PA previously treated with vehicle or TOIDC. **E** Abundance of lipogenesis-related mRNAs in HepG2 by OA/PA previously treated with vehicle or TOIDC and T0901317. **F** Immunoblots (proteins indicated) in vehicle-and TOIDC-treated primary hepatocytes stimulated by OA/PA previously. **G** Quantification of (**F**). **H** Immunoblots (proteins indicated) in vehicle-, TOIDC-and T0901317-treated primary hepatocytes stimulated by OA/PA previously. **I** Quantification of (**H**). **J** Immunoblots (proteins indicated) in vehicle-and TOIDC-treated HepG2 stimulated by OA/PA previously. **K** Quantification of (**J**). **L** Immunoblots (proteins indicated) in the vehicle, TOIDC, and T0901317 treated HepG2 stimulated by OA/PA previously. **M** Quantification of (**L**). Data are presented as mean ± SEM of three independent biological replicates; *P < 0.05; **P < 0.01; *ns* no significance

In order to explore the detailed mechanism of TOIDC on lipid accumulation in MAFLD, we treated primary hepatocytes with OA and PA together with TOIDC and vehicle. qPCR revealed that the expression of the genes involved in lipogenesis, including *Srebp1*, *Fasn*, *Acaca*, and *Scd1* were significantly down-regulated in TOIDC-treated hepatocytes (Fig. 4A). However, TOIDC treatment has little effect on the expression of lipid transport genes such as *CD36* and lipid oxidation genes such as *PPARα* and *CPT1α* (Fig. 4A). The Liver X receptor (LXR) is the transcriptional activator of the *Srebp1,* which upregulates the mRNA level of *SREBP1* [21]. Therefore, we hypothesized that TOIDC might be affecting lipogenesis genes through the LXR-SREBP axis. To test this hypothesis, we treated primary hepatocytes with an LXR agonist T0901317, and using Nile Red staining; we found that the TIOC-mediated lipid reduction lipid was disrupted by T0901317 (Fig. 4B). In accordance with these results, T0901317 treated group (TOIDC + T0901317) also showed significantly lower mRNA levels of *Srebp1* and its downstream genes, *Fasn* and *Acaca*, compared to TOIDC alone group (Fig. 4C). Suggesting that TOIDC is likely to ameliorate MAFLD through the lipogenesis pathway by downregulating the transcriptional levels of *Srebp1*. Consistent with the above results, we also found that TOIDC had a comparable effect on *SREBP1* and its downstream genes in human cells, HepG2 (Fig. 4D–E). To test whether TOIDC could down-regulate the abundance of lipogenesis-related proteins, we collected the protein from primary hepatocytes stimulated by a mixture of OA and PA treated with TOIDC or vehicle. Surprisingly, compared to the vehicle, TOIDC significantly reduced the protein abundance of SREBP1 and FASN (Fig. 4F–G). In addition, when T0901317 and TOIDC were added simultaneously in primary hepatocytes stimulated by a mixture of OA and PA, the previous effect was reversed. (Fig. 4H–I). Consistently, TOIDC showed the same function in HepG2 cells (Fig. 4J–M). Moreover, SREBP1 is regulated by activation of LXR and inactivation of FXR in the lipogenesis pathway. Our molecular dynamics simulation disclosed that TOIDC prefers to bind to the FXR structure, however TOIDC has no combination with LXRα (Additional file 2: Fig. S2A–C). In conclusion, these results indicated that TOIDC reduced lipid accumulation in cells through down-regulation of the SREBP1-dependent lipogenesis pathway.

### Administration of TOIDC in DIO mice curbs MAFLD and obesity

**Fig. 5:**
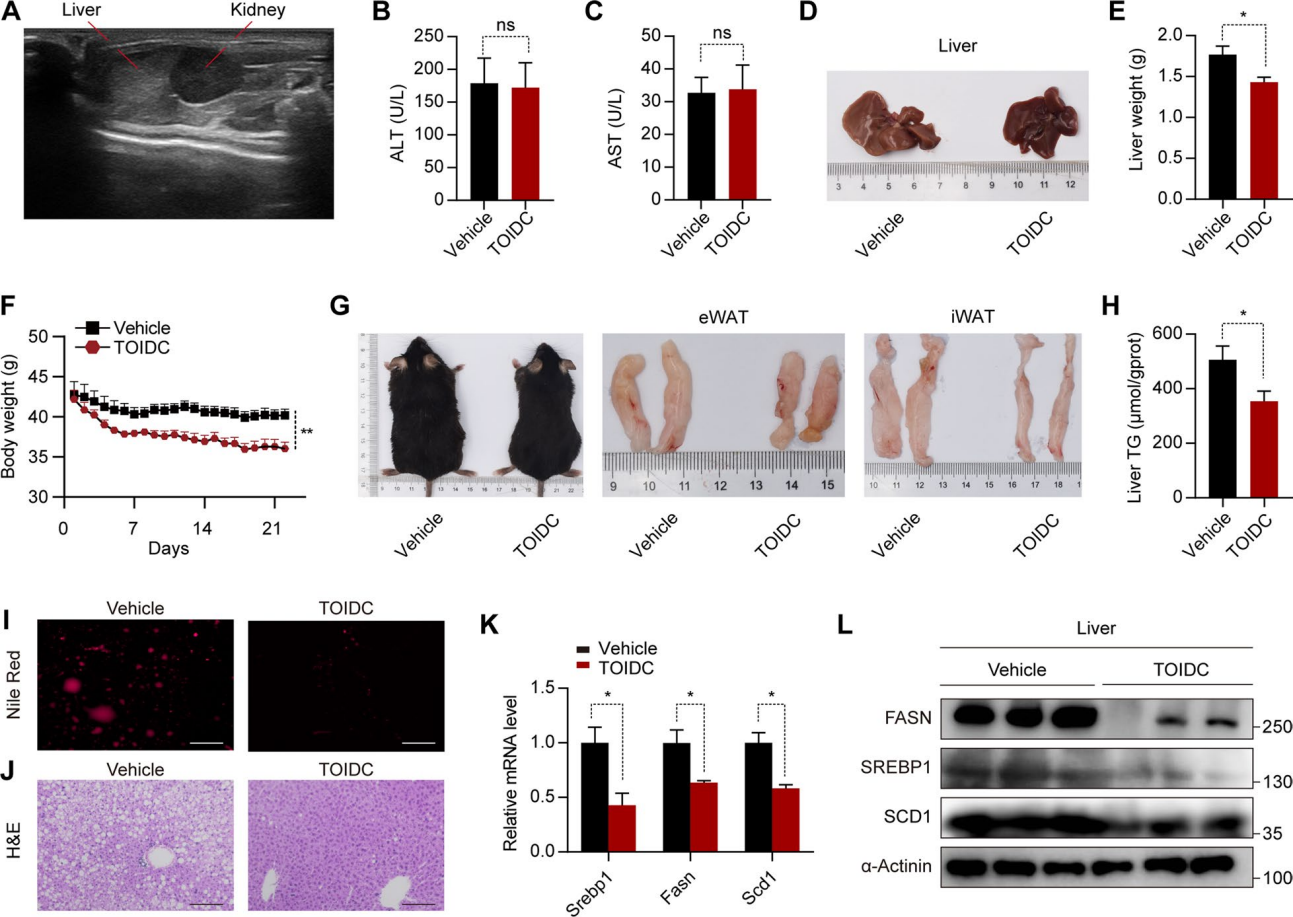
Administration of TOIDC in DIO mice curbs MAFLD and obesity (n = 5 in each group). **A** Ultrasound photo of diet-induced obese (DIO) mice before treatment. **B** The serum ALT activity of DIO mice administered with TOIDC or Vehicle. **C** The serum AST activity of DIO mice administered with TOIDC or Vehicle. **D** Representative of the livers of the vehicle or TOIDC treatment DIO mice. **E** Liver weight of the vehicle or TOIDC treatment DIO mice. **F** Body weight curve of DIO during TOIDC treatment mice. **G** Representative of DIO and fat pad photos of vehicle or TOIDC treatment DIO mice. **H** Triglyceride content in liver of vehicle or TOIDC treatment DIO mice. **I** Nile staining of liver tissue of vehicle or TOIDC treatment DIO mice. Scale bar, 100 µm. **J** H&E staining of liver tissue of vehicle or TOIDC treatment DIO mice. Scale bar, 100 µm. **K** The abundance of lipogenesis related mRNAs in the liver of DIO mice. **L** The abundance of lipogenesis related protein in the liver of DIO mice. Data are presented as mean ± SEM of three independent biological replicates; *P < 0.05; **P < 0.01; *ns* no significance

Next, we assessed the pharmacological potential of TOIDC in vivo*.* Firstly, we constructed DIO by feeding C57BL/6 J Mice with a HFD for 16 weeks and then confirmed the construction of the MAFLD model in vivo by ultrasound analysis (Fig. 5A). The MAFLD mice were randomly divided into two groups, with 5 mice in each group. According to the CCK8 assay in vitro, we detected that the TOIDC had certain toxicity at 60 µM. Therefore, MAFLD mice were treated daily with a relatively low dose (10 mg/kg) of TOIDC intraperitoneally (i.p.) for 3 weeks. Meanwhile, the mice in the control group were intraperitoneally injected with the same amount of DMSO. TOIDC treatment had little effect on serum levels of alanine aminotransferase (ALT) and aspartate aminotransferase (AST) in DIO mice, which indicated low hepatotoxicity of TOIDC (Fig. 5B–C). Moreover, analysis of the raw liver tissues showed that TOIDC treatment effectively reduced the size and weight of livers in DIO mice (Fig. 5D–E). During the experiment, we found a significant reduction in the body weight, body size, and fat content of the TOIDC-treated group compared with the control group (Fig. 5F–G), while no significant difference in the food intake was noticed between the two groups (Additional file 3: Fig. S3A). Additionally, we extracted the mRNA from subcutaneous fat of DIO mice and found the thermogenic genes is upregulated after TOIDC injection. (Additional file 3: Fig. S3B). Consistent with these results, the TOIDC-treated DIO mice also showed improved insulin resistance as determined by insulin tolerance tests, although no significant difference in glucose tolerance tests was reported (Additional file 3: Fig. 3C–F). Besides, the levels of hepatic TG of the TOIDC-treated group were significantly reduced (Fig. 5H). In addition, Nile Red staining and H&E staining of the liver slides unravel that TOIDC significantly suppressed lipid accumulation in the liver (Fig. 5I–J). Furthermore, we detected that the mRNA and protein levels of lipogenesis-related genes, including *Srebp1*, *Fasn*, and *Scd1*, were all reduced in the TOIDC-treated group compared to the control group (Fig. 5K–L), however we didn’t find the significant difference of lipid oxidation genes or lipolysis genes (Additional file 3: Fig. S3G). In addition, we observed that TOIDC ameliorates MAFLD with no significant effect on plasma triglyceride levels (Additional file 3: Fig. S3H). Collectively, these data showed that inhibition of SREBP1 by TOIDC ameliorates MAFLD in DIO mice.

## Discussion

MAFLD is a dysfunctional metabolic disease with increased TG in the liver along with the presence of at least one of three criteria that include overweight or obesity, T2DM or clinical evidence of metabolic dysfunction. Unfortunately, the majority of MAFLD patients are asymptomatic until the advanced stage, which puts patients at higher risk of carcinoma due to the lack of clinical drugs to inhibit the progression. Notably, MAFLD could be reversed more easily at an earlier period, so it is imperative to find an effective drug to inhibit the progression of MAFLD at an early stage [5, 6]. Adipose tissue decomposition, dietary nutrition intake, and de novo lipogenesis pathway are important ways for FFA accumulation [8]. Moreover, DNL not only increases FFAs but its intermediate products are also known to inhibit β-oxidation, further aggravating MAFLD [10].

In the developing field of innovating drugs, HCS is a new screening technology. The technique provides a large amount of relevant information from a single experiment and determines the biological activity and potential toxicity of candidate drugs [22]. We used HCS to find out that the small compound TOIDC is capable of suppressing FFAs-induced MAFLD in vitro. TOIDC reduces cumulative triglycerides in hepatocytes without cytotoxicity, while TOIDC fails to elevate oxygen consumption. Further experiments revealed that the inhibition of the lipogenesis pathway might be a reason for a decrease in cumulative triglycerides [9]. In addition, the improvement of whole-body metabolic dysfunction of TOIDC treatment in the DIO mice, especially MAFLD, for 3 weeks confirmed the anti-MAFLD function of TOIDC. These findings also approved the value of HCS for discovering more candidate compounds.

TOIDC is a novel small compound, which contains following characterization: Mp: 139–140 °C. ^1^H NMR (500 MHz, CDCl3) δ 7.53 (d, *J* = 7.4 Hz, 1H), 7.44 (td, *J* = 7.8, 1.0 Hz, 1H), 7.22 (t, *J* = 7.6 Hz, 1H), 7.00 (d, *J* = 7.9 Hz, 1H), 3.79 (s, 6H), 3.63 (s, 3H). ^13^C NMR (125 MHz, CDCl3) δ 193.7, 165.2, 145.9, 130.2, 128.0, 125.7, 124.8, 109.8, 75.1, 53.9, 31.8. HRMS (EI-TOF) m/z: [M] + calculated for C13H13NO4S 279.0565, found 279.0567.), 7.44 (td, *J* = 7.8, 1.0 Hz, 1H), 7.22 (t, *J* = 7.6 Hz, 1H), 7.00 (d, *J* = 7.9 Hz, 1H), 3.79 (s, 6H), 3.63 (s, 3H). ^13^C NMR (125 MHz, CDCl3) δ 193.7, 165.2, 145.9, 130.2, 128.0, 125.7, 124.8, 109.8, 75.1, 53.9, 31.8. HRMS (EI-TOF) m/z: [M] + calculated for C13H13NO4S 279.0565, found 279.0567. It has never been reported to treat dysfunctional metabolic diseases, although it has been revised from the core structure 2-thioxoindoline scaffold which has been reported in a variety of biological properties [23]. In addition, these compounds could serve as substance P antagonists [24–26], 5HT-3 serotonin receptor antagonists [27, 28], tyrosine kinase inhibitors [29, 30], antitumor agents [31, 32], and agrochemical fungicides [33]. Our study indicates that the TOIDC has a huge potential for metabolic benefits mediated by TOIDC-inhibited lipogenesis. According to the previous study, SREBP1 upregulates and activates FASN, ACACA, and SCD1, which as key enzymes regulate the lipogenesis pathway [34]. In this study, we found that TOIDC inactivates lipogenesis mainly through the SREBP1-dependent pathway. T0901317, a direct LXRα activator, induces the lipogenesis pathway by upregulating the mRNA level of SREBP1 (35). TOIDC also reverses the effect of T0901317, confirming that TOIDC ameliorates MAFLD mainly through down-regulating SREBP1. Moreover, SREBP1 is regulated by activation of LXR and inactivation of FXR in the lipogenesis pathway. Our molecular dynamics simulation disclosed that TOIDC prefers to bind to FXR structure. Moreover, in contrast to the previous reported compounds (e.g., Firsocostat), TOIDC ameliorates MAFLD without high risk of hyperglyceridemia. These results indicate that TOIDC may act as a valuable lead compound for MAFLD treatment.

In this study, we found that the small compound TOIDC obviously reduces triglyceride accumulation in the liver with a suitable dose without toxicity or hyperglyceridemia and TOIDC is likely to bind to the FXR structure. Considering that TOIDC can also mediate suppression of accumulative triglycerides in the liver, it can also prove beneficial for future studies to consolidate the hypothesis and find specific targets which might bring new prospects for drug development. Furthermore, for achieving higher and more specific binding affinity, necessarily, TOIDC could be structural revised optimally or used for finding compounds alike.

## Conclusion

Our study identified a novel small compound which ameliorates MAFLD effectively and down-regulates lipogenesis pathway dependent on SREBP1. Furthermore, we emphasize the available of the advanced technique, HCS, to find more lead small compounds and the drug development could be further stepped in discovery.

## Supplementary Information

**Additional file 1: Fig. S1**. Compounds with similar structures have no effect on reducing lipid accumulation. **A** Fluorescent images representing the compounds whose structure is similar to TOIDC, and with effect on the lipid accumulation through high-content screening analysis. Scale bar, 200 µm. **B** Fluorescent images representing the compounds whose structure is similar to TOIDC and with toxicity. Scale bar, 100 µm.

**Additional file 2: Fig. S2**. FXR is a potential target of TOIDC. **A** Representative images of auto-docking for FXR and TOIDC. **B** Protein structure of FXR. **C** Representative images of auto-docking for LXRα and TOIDC.

**Additional file 3: Fig. S3**. TOIDC improves obesity-associated metabolic dysfunction in mice. **A** Food intake of diet-induced obese (DIO) mice administered with TOIDC or Vehicle. **B** The abundance of thermogenic and lipolysis related mRNAs in iWAT of DIO mice. **C** Glucose tolerance tests (GTT) performed in DIO mice (i.p. injection of glucose, 1.5 g/kg) fasted 16 h after 3-week treatment with TOIDC or vehicle. (n = 5 for each treatment). **D** Area under curve (AUC) for glucose based on data in B. **E** Insulin tolerance tests (ITT) performed in DIO mice (i.p. injection of insulin, 0.75 U/kg) fasted 4 h after 3-week treatment with TOIDC or vehicle. **F** Area under curve (AUC) for glucose based on data in D. **G** The abundance of lipid oxidation and lipolysis related mRNAs in liver of DIO mice. **H** Triglyceride content in plasm of vehicle or TOIDC treatment DIO mice. Data are presented as mean ±SEM of three independent biological replicates; *P < 0.05; **P < 0.01; *ns* no significance.

**Additional file 4: Table S1**. Structure of compounds.

**Additional file 5: Table S2**. qPCR primer sequences.

## Data Availability

The datasets used and/or analyzed during the current study are available from the corresponding author on reasonable request.

## Abbreviations

MAFLD: Metabolic-associated fatty liver disease
TG: Triglycerides
T2DM: Type 2 diabetes mellitus
FFA: Free fatty acid
DNL: De novo lipogenesis
SREBP: Sterol regulatory element-binding protein
ACACA: Acetyl-CoA carboxylase
FASN: Fatty acid synthesis
HCS: High-content screening
TOIDC: Dimethyl 1-methyl-2-thioxoindoline-3,3-dicarboxylate
DMSO: Dimethyl sulfoxide
SPF: Specific pathogen free
DIO: Diet-induced obese
HFD: High-fat diet
ALT: Alanine aminotransferase
AST: Aspartate aminotransferase
PBS: Phosphate-buffered saline
PFA: Paraformaldehyde
GTT: Glucose tolerance test
ITT: Insulin tolerance tests
FBS: Fetal bovine serum
OA: Oleic acid
PA: Palmitic acid
RIPA: Radioimmunoprecipitation assay
BSA: Bovine serum albumin
FXR: Farnesoid X receptor
SEM: Standard errors of the means
LXR: Liver X receptor
