# Supplementary figures and images for: A novel small compound TOIDC suppresses lipogenesis via SREBP1-dependent signaling to curb MAFLD
Source: Nutr Metab (Lond). 2022 Dec 6;19:80. doi: 10.1186/s12986-022-00713-0 (PMC9727880; doi:10.1186/s12986-022-00713-0)

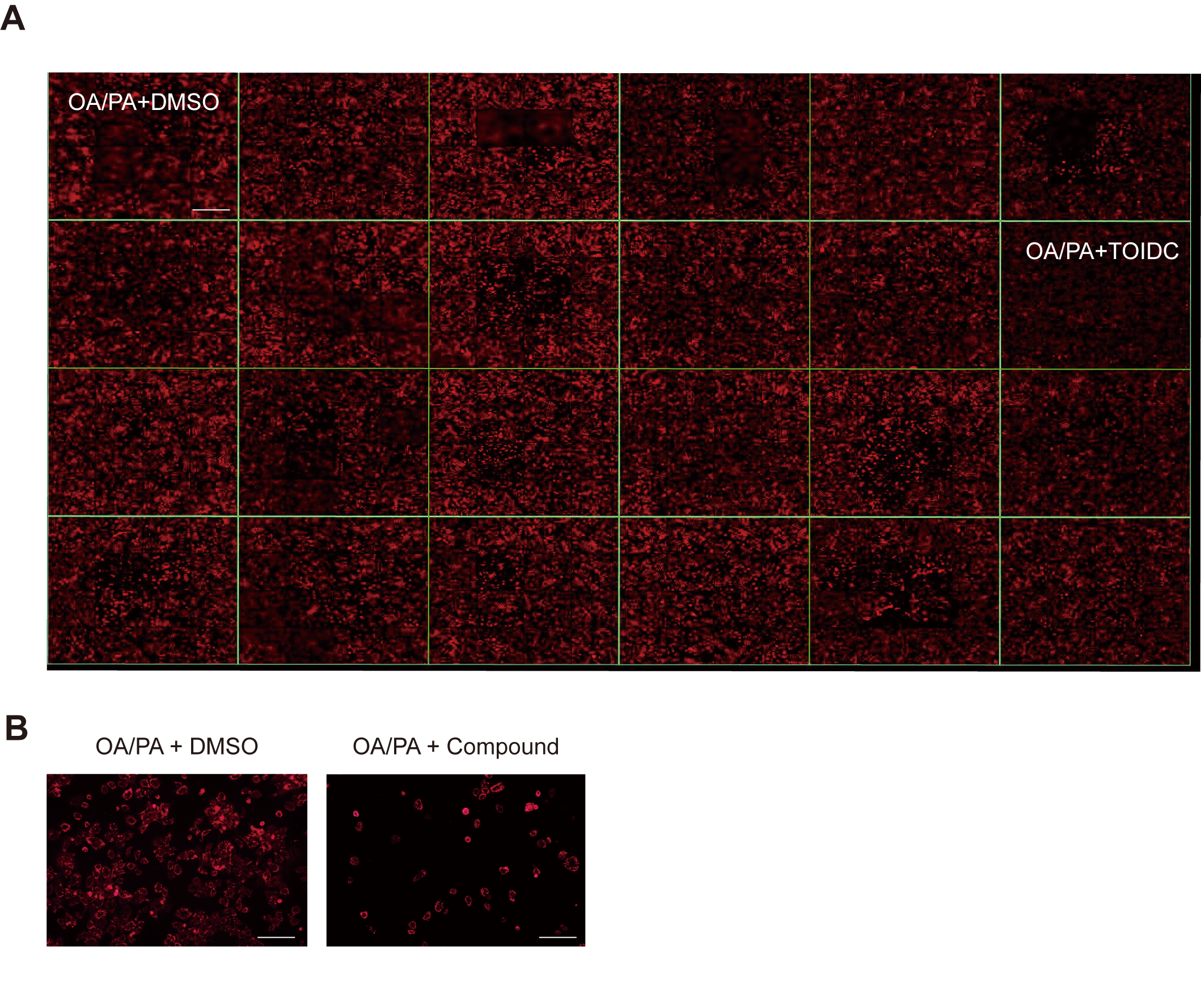

Supplement: Supplementary file 1 — Additional file 1: Fig. S1. Compounds with similar structures have no effect on reducing lipid accumulation. A Fluorescent images representing the compounds whose structure is similar to TOIDC, and with effect on the lipid accumulation through high-content screening analysis. Scale bar, 200 µm. B Fluorescent images representing the compounds whose structure is similar to TOIDC and with toxicity. Scale bar, 100 µm. [file 12986_2022_713_MOESM1_ESM.tif]

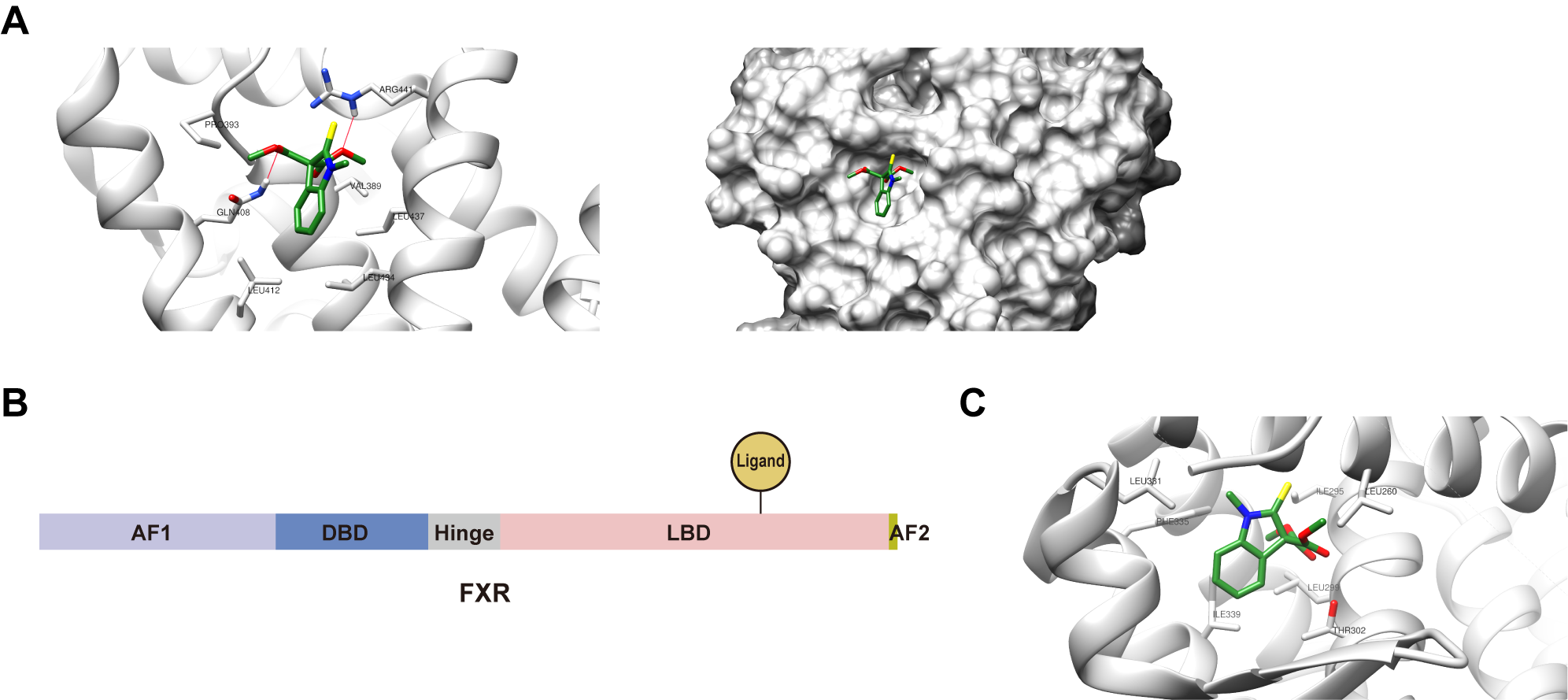

Supplement: Supplementary file 2 — Additional file 2: Fig. S2. FXR is a potential target of TOIDC. A Representative images of auto-docking for FXR and TOIDC. B Protein structure of FXR. C Representative images of auto-docking for LXRα and TOIDC. [file 12986_2022_713_MOESM2_ESM.tif]

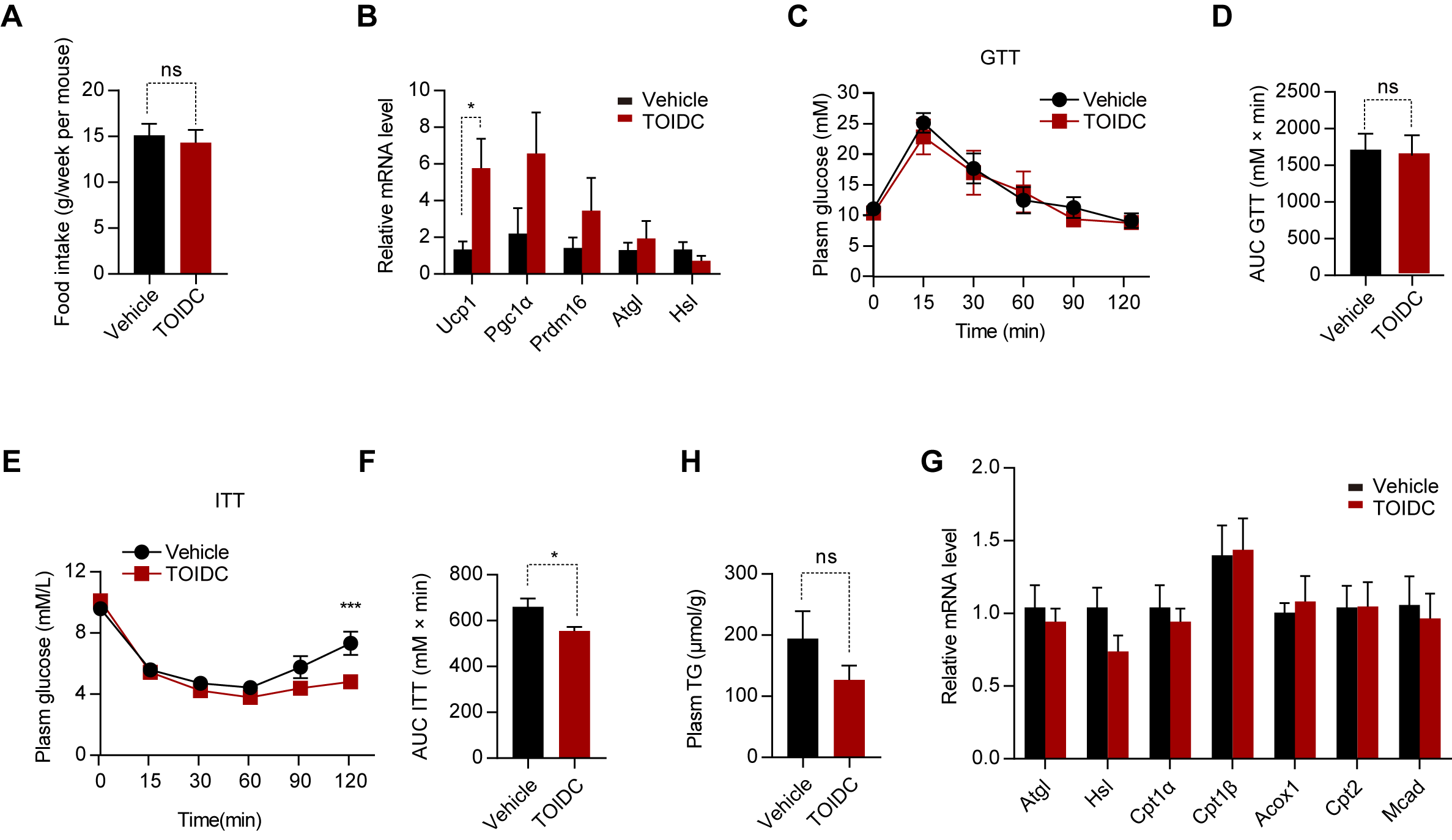

Supplement: Supplementary file 3 — Additional file 3: Fig. S3. TOIDC improves obesity-associated metabolic dysfunction in mice. A Food intake of diet-induced obese (DIO) mice administered with TOIDC or Vehicle. B The abundance of thermogenic and lipolysis related mRNAs in iWAT of DIO mice. C Glucose tolerance tests (GTT) performed in DIO mice (i.p. injection of glucose, 1.5 g/kg) fasted 16 h after 3-week treatment with TOIDC or vehicle. (n = 5 for each treatment). D Area under curve (AUC) for glucose based on data in B. E Insulin tolerance tests (ITT) performed in DIO mice (i.p. injection of insulin, 0.75 U/kg) fasted 4 h after 3-week treatment with TOIDC or vehicle. F Area under curve (AUC) for glucose based on data in D. G The abundance of lipid oxidation and lipolysis related mRNAs in liver of DIO mice. H Triglyceride content in plasm of vehicle or TOIDC treatment DIO mice. Data are presented as mean ±SEM of three independent biological replicates; *P < 0.05; **P < 0.01; ns no significance. [file 12986_2022_713_MOESM3_ESM.tif]
