# Supplementary material for: A novel small compound TOIDC suppresses lipogenesis via SREBP1-dependent signaling to curb MAFLD
Source: Nutr Metab (Lond). 2022 Dec 6;19:80. doi: 10.1186/s12986-022-00713-0 (PMC9727880; doi:10.1186/s12986-022-00713-0)
Supplement: Supplementary file 4 — Additional file 4: Table S1. Structure of compounds. [file 12986_2022_713_MOESM4_ESM.docx]

**Supplementary Table 1. Structure of compounds**

| **Number** | **Structure** |
| --- | --- |
| Yz-2a |  |
| Yz-2b |  |
| Yz-2c |  |
| Yz-2d |  |
| Yz-2e |  |
| Yz-2f |  |
| Yz-2g |  |
| Yz-2h |  |
| Yz-2i |  |
| Yz-2j |  |
| Yz-2k |  |
| Yz-2l |  |
| Yz-2m |  |
| Yz-2n |  |
| Yz-2o |  |
| Yz-2p |  |
| Yz-2q |  |
| Yz-2r |  |
| Yz-2s |  |
| Yz-2t |  |
| Yz-2u |  |
| Yz-2v |  |
| Yz-2w |  |
| Yz-2x |  |
| Yz-2y |  |
| Yz-2z |  |
| Yz-2aa |  |
| Yz-2ab |  |
| Yz-2ac |  |
| Yz-2ad |  |
| Yz-2ae |  |
| Yz-2af |  |
| Yz-2ag |  |
| Yz-2ah |  |
| Yz-2ai |  |
| Yz-2aj |  |
| Yz-2ak |  |
| Yz-2al |  |
| Yz-3a |  |
| Yz-3b |  |
| Yz-3c |  |
| Yz-3d |  |
| Yz-3e |  |
| Yz-3f |  |
| Yz-3g |  |
| Yz-3h |  |
| Yz-3i |  |
| Yz-3j |  |
| Yz-3k |  |
| Yz-3l |  |
| Yz-3m |  |
| Yz-3n |  |
| Yz-3o |  |
| Yz-3p |  |
| Yz-3q |  |
| Yz-3q’ |  |
| Yz-3r |  |
| Yz-3r’ |  |
| Yz-3s |  |
| Yz-3s’ |  |
| Yz-3t |  |
| Yz-3t’ |  |
| Yz-3u |  |
| Yz-3u’ |  |
| Yz-3v |  |
| Yz-3w |  |
| Yz-3x |  |
| Yz-3y |  |
| Yz-3z |  |
| Yz-3aa |  |
| Yz-3ab |  |
| Yz-3ac |  |
| Yz-3ad |  |
| Yz-3ae |  |
| Yz-3af |  |
| Yz-4 |  |
| Yz-4 |  |
| Yz-4 |  |
| Yz-4 |  |
| Yz-4 |  |
| Yz-4 |  |
| Yz-4 |  |
| Yz-4 |  |
| Yz-4 |  |
| Yz-4 |  |
| Yz-4 |  |
| Yz-4 |  |
| Yz-4 |  |
| Yz-4 |  |
| Yz-4 |  |
| Yz-4 |  |
| Yz-4 |  |
| Yz-4 |  |
| Yz-4 |  |
| Yz-4 |  |
| Yz-4 |  |
| Yz-4 |  |
| Yz-4 |  |
| Yz-4 |  |
| Yz-4 |  |
| Yz-4 |  |
