# Supplementary material for: A novel small compound TOIDC suppresses lipogenesis via SREBP1-dependent signaling to curb MAFLD
Source: Nutr Metab (Lond). 2022 Dec 6;19:80. doi: 10.1186/s12986-022-00713-0 (PMC9727880; doi:10.1186/s12986-022-00713-0)
Supplement: Supplementary file 5 — Additional file 5: Table S2. qPCR primer sequences. [file 12986_2022_713_MOESM5_ESM.docx]

**Supplementary Table 2. qPCR primer sequences**

| **Gene** | **Sequence** |
| --- | --- |
| ***Srebp1*** |  |
| Forward | GGAGCCATGGATTGCACATT |
| Reverse | GGCCCGGGAAGTCACTGT |
| ***Fasn*** |  |
| Forward | GGCTGCGTGGCTATGATTATGG |
| Reverse | TGTAGACTCTGCTGGCTAGAACC |
| ***Acaca*** |  |
| Forward | ATGGGCGGAATGGTCTCTTTC |
| Reverse | TGGGGACCTTGTCTTCATCAT |
| ***Scd1*** |  |
| Forward | TGCGATACACTCTGGTGCTCAA |
| Reverse | AAGGTGTGGTGGTAGTTGTGGAA |
| ***Cd36*** |  |
| Forward | ATGGGCTGTGATCGGAACTG |
| Reverse | GTCTTCCCAATAAGCATGTCTCC |
| ***Pparα*** |  |
| Forward | AGAGCCCCATCTGTCCTCTC |
| Reverse | ACTGGTAGTCTGCAAAACCAAA |
| ***Cpt1α*** |  |
| Forward | CTCCGCCTGAGCCATGAAG |
| Reverse | CACCAGTGATGATGCCATTCT |
| ***SREBP1*** |  |
| Forward | TGAGGACAGCAAGGCAAAGC |
| Reverse | GCAGGACAGGCAGAGGAAGA |
| ***FASN*** |  |
| Forward | CACACTCACCAGCAACACCAA |
| Reverse | ACTTCCTTTCTCTTCACCCAAACA |
| ***ACACA*** |  |
| Forward | ACCTGCGAGTAGAGACACAATTC |
| Reverse | TTCTTGGTGACTTGAGCGTGAG |
| ***SCD1*** |  |
| Forward | TGGCTTGCTGATGATGTGCTT |
| Reverse | AGGAGTGGTGGTAGTTGTGGAA |
